# Alcalase Microarray Base on Metal Ion Modified Hollow Mesoporous Silica Spheres as a Sustainable and Efficient Catalysis Platform for Proteolysis

**DOI:** 10.3389/fbioe.2020.00565

**Published:** 2020-06-10

**Authors:** Qi Zeng, Qi Li, Di Sun, Mingming Zheng

**Affiliations:** Oil Crops Research Institute, Chinese Academy of Agricultural Sciences, Key Laboratory of Oilseeds Processing, Ministry of Agriculture, Hubei Key Laboratory of Lipid Chemistry and Nutrition, Wuhan, China

**Keywords:** alcalase microarray, hollow mesoporous silica spheres (HMSS), metal ion modified nanocomposite, metal-protein affinity, alcalase immobilization, proteolysis

## Abstract

The industrial exploitation of protease is limited owing to its sensitivity to environmental factors and autolysis during biocatalytic processes. In the present study, the alcalase microarray (*Bacillus licheniformis*, alcalase@HMSS-NH_2_-Metal) based on different metal ions modified hollow mesoporous silica spheres (HMSS-NH_2_-Metal) was successfully developed *via* a facile approach. Among the alcalase@HMSS-NH_2_-Metal (Ca^2+^, Zn^2+^, Fe^3+^, Cu^2+^), the alcalase@HMSS-NH_2_-Fe^3+^ revealed the best immobilization efficiency and enzymatic properties. This tailor-made nanocomposite immobilized alcalase on a surface-bound network of amino-metal complex bearing protein-modifiable sites *via* metal-protein affinity. The coordination interaction between metal ion and alcalase advantageously changed the secondary structure of enzyme, thus significantly enhanced the bioactivities and thermostability of alcalase. The as-prepared alcalase@HMSS-NH_2_-Fe^3+^ exhibited excellent loading capacity (227.8 ± 23.7 mg/g) and proteolytic activity. Compared to free form, the amidase activity of alcalase microarray increased by 5.3-fold, the apparent kinetic constant V_max_/K_m_ of alcalase@HMSS-NH_2_-Fe^3+^ (15.6 min^−1^) was 1.9-fold higher than that of free alcalase, and the biocatalysis efficiency increased by 2.1-fold for bovine serum albumin (BSA) digestion. Moreover, this particular immobilization strategy efficiently reduced the bioactivities losses of alcalase caused by enzyme leaking and autolysis during the catalytic process. The alcalase microarray still retained 70.7 ± 3.7% of the initial activity after 10 cycles of successive reuse. Overall, this study established a promising strategy to overcome disadvantages posed by free alcalase, which provided new expectations for the application of alcalase in sustainable and efficient proteolysis.

## Hightlights

- Fabrication of alcalase microarray based on metal-protein affinity.
- Alcalase@Fe^3+^ revealed much better performance than Ca^2+^, Zn^2+^and Cu^2+^.
- Proteolysis activity of immobilized alcalase increased 2.1-fold than that of free one.
- Activation of alcalase by coordination with metal ions was confirmed by FT-IR.
- The alcalase microarray still retained 70.7% of activity after 10 cycles.

## Introduction

Protease, like alcalase, is notable for its high biocatalytic activity and extensive adaptability in various fields (Jin et al., 2010; Zhang et al., 2018). However, the industry application of protease is hampered by its low operational stability, difficulties to recover and autolysis behavior (Öztürk et al., 2012). Benefiting from the development of modern biotechnology, enzyme immobilization provides a straightforward way to overcome these drawbacks (Bernal et al., 2018b). Immobilizing the enzyme on solid materials could enhance enzyme stability and enabled enzyme to reuse. Moreover, the autolysis posed by free protease could also be inhibited (Zhou and Hartmann, 2013). So far, there are four main methods for enzyme immobilization, such as physical adsorption, covalent binding, cross-linking of enzymes and entrapment (Xiao et al., 2018). Unfortunately, those conventional immobilization methods (e.g., physical adsorption, covalent binding, etc.) would lead to the losses of enzyme biocatalytic activity inevitably due to enzyme leakage and/or partial denaturation (Zhou and Hartmann, 2013). In this context, an efficient immobilization method should be developed which balance among catalytic activity, operational stability and economic applicability.

The concept of protein affinity for metal ions is well-known in the form of immobilized metal (ion) affinity chromatography (IMAC). In recent years, the application of metal-protein affinity has been extended to many fields except separation of protein. In particular, the potential application of metal-protein affinity in enzyme immobilization has gradually attracted extensive attention of researchers. Different from traditional immobilization method, this novel enzyme immobilization method was based on the interaction between electron donor groups (carboxy, amino, imidazole and thiol groups, etc.) present on protein surface and metal ions which secured on matrix by multidentate chelators (Ueda et al., 2003). Therefore, different combinations of metal ions and multi-dentate chelates could be used to obtain functional carriers with different affinity to target enzymes (Torres et al., 2004). It should be noted that the final structure of metal ions which chelated with the chelating group must contain free coordination sites for adsorption or binding with enzymes (Ueda et al., 2003). In many cases, the pre-coordination of metal ions with multidentate chelators could not only reduce the adverse effects of metal ions for enzyme structure but also improve the biocatalytic activity and operational stability of enzyme through stabilizing conformation of its catalytic sites (Wang et al., 2019). On the other hand, metal-protein affinity based on multiple forces including electrostatic interaction and metal-organic coordination could reduce the leaching of immobilized enzymes due to environmental factors, thereby retaining the biocatalytic activity during processing (Lai and Lin, 2018). Therefore, metal-protein affinity immobilization as a high-efficiency and sustainable procedure has a broad application prospect in enzyme immobilization.

In addition to proper immobilization method, suitable supports for targeted enzyme was also essential for immobilization (Dong et al., 2019b; Jankowska et al., 2019). In order to gain higher enzyme loading capacity and biocatalytic activity, it is always highly desirable to invent novel supports with high-performance (Antecka et al., 2018; Elizarova and Luckham, 2018; Zdarta et al., 2019; Jankowska et al., 2020). Recently, Hollow Mesoporous Silica Spheres (HMSS) have inspired prominent research interests due to their fascinating properties including high surface areas, ordered mesopores and abundant modifiable site on surface (Teng et al., 2019). To be specific, the highly specific surface area of HMSS provided an essential prerequisite for efficient mass transfer during the catalysis process (Hartmann and Kostrov, 2013). In addition, uniform and ordered mesopores of HMSS constructed a proper microenvironment for enzyme, which could increase the enzyme stability and reduce denaturation of enzyme (Hartmann and Jung, 2010). Besides, the abundant modifiable sites on HMSS surface were allowed to design specific surface properties for targeted enzyme. Up to now, HMSS had been successfully used in immobilization of lipases and alcalase (Ibrahim et al., 2016; Dong et al., 2019a).

Herein, we reported a novel alcalase microarray based on metal ions modified amino-functionalized HMSS as a sustainable and efficient catalysis platform for proteolysis. HMSS was utilized as matrix, (3-aminopropyl) trimethoxy silane (APTMS) was used to graft amino group on HMSS surface. Then, the terminal amino groups on HMSS-NH_2_ surface worked as linkers to anchor metal ions, thus alcalase could be immobilized on a surface-bound network of amino-metal complex bearing protein-modifiable sites. Among different alcalase@HMSS-NH_2_-Metal (Zn^2+^, Ca^2+^, Fe^3+^, Cu^2+^), the alcalase@HMSS-NH_2_-Fe^3+^ revealed the best immobilization efficiency, enzymatic properties and operational stability. Overall, this study established a promising strategy to overcome disadvantages posed by free alcalase, which provided new expectations for the application of alcalase in sustainable and efficient proteolysis.

## Materials and Methods

### Materials

Alcalase 2.4 L (protease from *Bacillus licheniformis*) (>2.4 U/g solution) was obtained from the Novozymes, (3-aminopropyl) trimethoxy silane (APTMS), N-benzoyl-L-arginine ethylester (BAEE), fluorescein isothiocyante (FITC) and cetyltrimethylammonium bromide (CTAB) were purchase from Sigma-Aldrich. Toluene, acetone and sodium metasilicate nonahydrate (Na_2_SiO_3_·9H_2_O), ethanol and other chemicals were supplied by Sinopharm Chemical Reagent (Shanghai, China) and were of analytical reagent grade. Albumin from bovine serum (BSA), O-Phthalaldehyde (OPA), L-arginine, dithiothreitol (DTT) and bicinchoninic acid (BCA) Protein Assay Kit were obtained from Shanghai Yuan Ye biotechnology. Purified water was obtained with a Milli-Q apparatus (Millipore, Bedford, MA, USA).

### Preparation of Metal Ion Modified Amino-Functionalized Mesoporous Silica Spheres (HMSS-NH_2_-Metal)

The method of HMSS synthesis and purified was according to previous reports with slightly modified (Zheng et al., 2015; Dong et al., 2019a). The detailed prepared process of HMSS was described below: 78.0 g CTAB and 92.0 g solid Na_2_SiO_3_·9H_2_O were added into 1,200 mL ultrapure water, and stirred vigorously at 30°C until the solution became clear. Subsequently, 75 mL ethyl acetate was quickly added into the clear solution with 30 s vigorous stirring. Then, the mixture was standing for 5 h at 30°C without any stirring. Next, the mixture solution was aged at 90°C for 48 h. Then, the mixture solution was filtered to gain the solid products and dried at room temperature. Finally, the dried HMSS was calcined at 550°C for 5 h in the muffle furnace and stored dry for further use. At the next stage, HMSS nanoparticles were modified by (3-aminopropyl) trimethoxy silane (APTMS) grafting amino group on HMSS surface (Bernal et al., 2018a). 1.0 g of native HMSS was added in contact with 30 mL of toluene at 10% of APTMS. After the reflux at 105 °C for 6 h, the material was filtered and washed with water-acetone solution. Subsequently, metal ion ligand anchor on HMSS-NH_2_ surface was performed as described previously with minor modification (Yang et al., 2010). Briefly, 1.0 g HMSS-NH_2_ was directly adding in a 25 mL ethyl alcohol which containing 1.5 mmol of different metal ions (CaCl_2_, ZnCl_2_, FeCl_3_, CuCl_2_), the mixture was stirred under a nitrogen atmosphere for 48 h at room temperature, the final products were filtered washed with acetone and ethyl alcohol, and air-dried for 24 h. The nanoparticles denoted as HMSS-NH_2_-Ca^2+^, HMSS-NH_2_-Zn^2+^, HMSS-NH_2_-Fe^3+^, HMSS-NH_2_-Cu^2+^ according to the different metal ions contained in the solution.

### Immobilization of Alcalase on HMSS-NH_2_-Metal

Firstly, 25 mL alcalase solution (Alcalase 2.4L) was incubated at 4°C for 15 min, 200.0 mg HMSS-NH_2_-Metal was added into 10 mL ultrapure water and dispersed with ultrasound assist for 3 min. Then, the solution which dispersed 200.0 mg HMSS-NH_2_-Metal was directly injected into alcalase solution, mild magnetic agitation at 4°C for 30 min. Subsequently, the mixture was centrifuged (10,000 rpm, 10 min) and the precipitate was washed three times by ultrapure water. The enzyme solution protein content before and after immobilization was determined by bicinchoninic acid (BCA) assay. The resulting alcalase@HMSS-NH_2_-Metal was lyophilized and stored at 4°C prior to use.

### Stability and Activity Evaluation of Immobilized Alcalase

Equal units of amidase activity and same protein content of free and immobilized alcalase were used in all assays.

N-benzoyl-L-arginine ethylester (BAEE) was used as substrate to evaluate the amidase activity of free alcalase and immobilized alcalase under the same conditions (25°C, pH 7.5). The hydrolytic cleavage of the easter linkage in BAEE could be catalyzed by alcalase, the produce N-α-benzoyl-L-arginine (BA) could be quantified by UV spectrometer (UV-1900, Shimadzu) at 253 nm. The calculation of enzymatic activity was shown in the following equation (Zhang et al., 2018):

(1) $$\begin{array}{r} U=\frac{\Delta A_{253}}{0.001\cdot G_{E}\cdot T} \end{array}$$

where U is the enzymatic activity of alkaline protease (U/mg), ΔA_253_ is the absorbance changed at 253 nm, GE is the amount of alcalase (free alcalase, alcalase@HMSS-NH_2_-Metal, mg), T is the reaction time (min).

The effects of different pH values and temperature on the activity of free alcalase and immobilized alcalase were studied by previous BAEE method. The 50 mM phosphate buffer (pH 6.5–9.0) was used to evaluate the effect of different pH on the activity of free alcalase and immobilized alcalase with same protein content. Thermal stability was determined incubating the immobilized enzyme (50 mM phosphate buffer, pH 7.5) at 85°C for 30, 60, 90, 120, and 150 min. The residual activity was determined and compared with the initial activity at assayed temperature.

In addition, the reusability of alcalase@HMSS-NH_2_-Fe^3+^ and alcalase@HMSS-NH_2_ was also evaluated with BAEE method. Briefly, alcalase@HMSS-NH_2_-Fe^3+^ and alcalase@HMSS-NH_2_ was dispersed into 2 mL of phosphate buffer (50 mM, pH 7.5). Then, 1 mL of DTT (30 mM) was added to the previous mixture and incubated at 40°C for 10 min. Subsequently, 3 mL of BAEE (10 mM) was added to the above mixture and incubated at 40°C for 5 min. Upon completion of one cycle, the alcalase microarray was separated by centrifugation and taken out directly. Then, the recovered alcalase@HMSS-NH_2_-Fe^3+^and alcalase@HMSS-NH_2_ were rinsed three times with 50 mM phosphate buffer (pH 7.5) to remove residual substrate. Next, the recover alcalase microarray was resuspended in a fresh reaction mixture. The residual enzyme activity of each cycle was calculated by taking the initial activity as 100%.

### Characterization of HMSS Carrier and Immobilized Alcalase

The surface morphologies of the samples were observed by scanning electron microscopy (SEM; SU8010, Hitachi, Tokyo, Japan) at 200 kV. Microstructure and compositional distribution of synthetic nanoparticles were investigated by STEM coupled with energy dispersive X-ray spectroscopy (EDX) elemental map (TEM; TECNAI G2 20S-TWIN, Hillsboro, OR, USA). Fourier transform infrared (FT-IR) spectra of synthetic nanoparticles were certified on an FTIR spectrometer (Bruker, Karlsruhe, Germany). Fluorescence confocal laser scanning microscopy (CLSM) images were attained on an Olympus IX71-A12FL and Nikon A1 confocal microscope (excitation wavelength = 488 nm).

### Determination of Enzymatic Kinetic Parameters

Serial concentration of BAEE (0.10, 0.08, 0.05, 0.02, 0.01, 0.008, 0.005 mol/L) were used to evaluate the catalytic activity of free alcalase and alcalase@HMSS-NH_2_-Fe^3+^. The enzymatic activity in this study was in line with Michaelis-Menten mechanism. The fundamental kinetic characteristics were calculated by the Michaelis-Menten constant (K_m_) and maximum reaction velocity (V_max_). The kinetic parameters K_m_ and V_max_ were determined by plotting Lineweaver-Burk double reciprocal plot and the initial rates were obtained by plotting concentration vs. time. The kinetic parameters are calculated from the following equation:

(2) $$\begin{array}{r} \frac{1}{V}=\frac{K_{m}}{V_{\max}}\cdot \frac{1}{C}+\frac{1}{V_{\max}}\; \end{array}$$

Where V and C are the initial reaction rates (mol/(L·min)) and initial concentration of BAEE (mol/L), respectively.

### Proteolysis Activity Evaluation of Free and Immobilized Alcalase

The BSA was dissolved in 50 mM phosphate buffer (pH 7.5) at 10.0 mg/mL concentration and used in this study. Free alcalase was in aqueous form, alcalase@HMSS-NH_2_-Fe^3+^, alcalase@HMSS-NH_2_ were completely dispersed in distilled water (80 mg protein equivalent/mL) before use. The catalysts with same protein equivalent was added to 10 mL BSA solution and incubated at 40°C for 0, 20, 40, 60, 80 min. At the end of incubation, the samples were heated at 100°C for 15 min to inactivate the enzyme. The degree of hydrolysis (DH) was determined by the OPA method according to the previous report (Nielsen et al., 2001). At last, the sodium dodecyl sulfate-polyacrylamide gel electrophoresis (SDS-PAGE) was used to analyze the molecular weight distribution of the hydrolysate produced by catalysts, the electrophoretogram was analysis by ChemiDoc^TM^ XRS^+^ system (Bio-Rad, USA).

## Results and Discussion

### Characterization of the HMSS Carriers and Immobilized Alcalase

Metal ions, especially transition metal ions, could form coordination complexes easily due to their underfilled d-orbit (Ivanova and Spiteller, 2017). In this study, Ca^2+^, Zn^2+^, Fe^3+^, and Cu^2+^ were selected to pre-coordination with HMSS-NH_2_. As shown in Table 1, HMSS-NH_2_ modified by Fe^3+^ exhibited the highest loading capacity for alcalase, up to 227.8 ± 23.7 mg/g. This result could be explained by principles of Lewis acids and bases theory (Lai and Lin, 2018): the electron donor groups present on enzyme surface were classified as Lewis base, and the metal ions were classified as Lewis acid, the loading capacity of pre-coordination metal ions for enzyme was governed by the strength and number of coordinate covalent bonds formed between enzyme and metal ions. In other words, the metal ions contained more residual coordination site after pre-coordination possess stronger loading capacity for the target enzyme. Although all those metal ions (Ca^2+^, Zn^2+^, Fe^3+^, and Cu^2+^) had six theoretical coordination sites, but Fe^3+^ had a larger atomic radius than other metal ions, which could bate the steric hindrance during the metal-protein binding process (Dunn et al., 2006). Amidase activity was commonly used to evaluate the hydrolysis ability of protease to peptide bonds (-CO-NH-). In this study, alcalase@HMSS-NH_2_-Fe^3+^ showed the highest amidase activity among all the metal ions. The increase of amidase activity could be attributed to the activation effect of pre-coordinated Fe^3+^ for alcalase.

**Table 1:** The result of HMSS-NH_2_ modified with different metal ion for alcalase immobilization.

**Category**	**Immobilization method**	**Loading capacity (mg/g)**	**Amidase activity (U/mg)**
Free Alcalase	—	—	10.5 ± 2.7
Alcalase@HMSS-NH_2_	Physical adsorption	214.0 ± 34.6	34.7 ± 2.4
Alcalase@HMSS-NH_2_-Ca^2+^	Metal-protein affinity	182.6 ± 27.6	28.7 ± 4.1
Alcalase@HMSS-NH_2_-Zn^2+^	Metal-protein affinity	124.5 ± 16.4	20.4 ± 2.6
Alcalase@HMSS-NH_2_-Cu^2+^	Metal-protein affinity	136.7 ± 38.1	22.7 ± 3.9
Alcalase@HMSS-NH_2_-Fe^3+^	Metal-protein affinity	227.8 ± 23.7	55.5 ± 6.2

**Figure 1:**
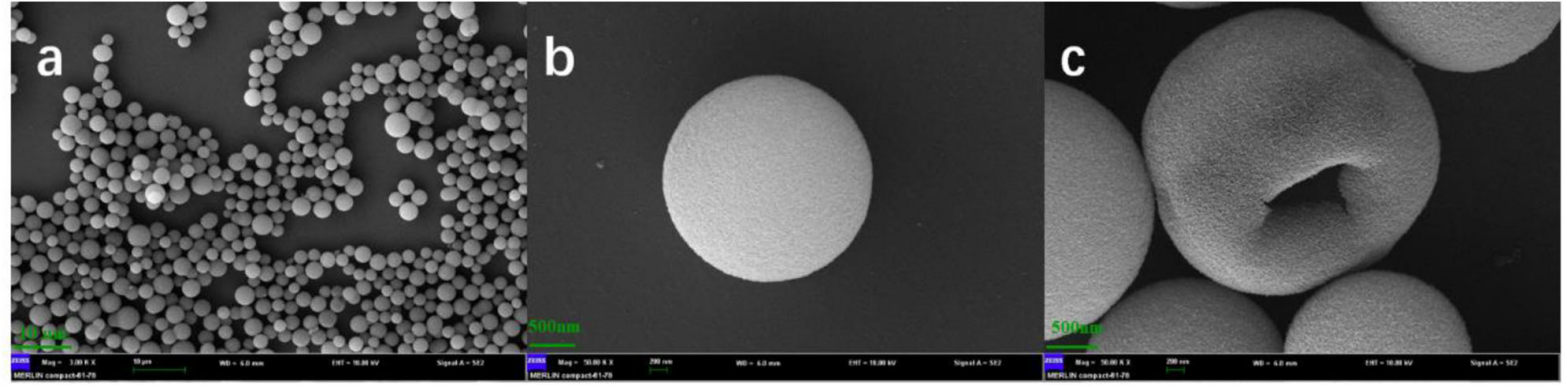
SEM images of HMSS-NH_2_ with different magnification. **(a)**: Mag = 3.0 kx, 10 μm, **(b,c)**: Mag = 50 kx, 200 nm.

**Figure 2:**
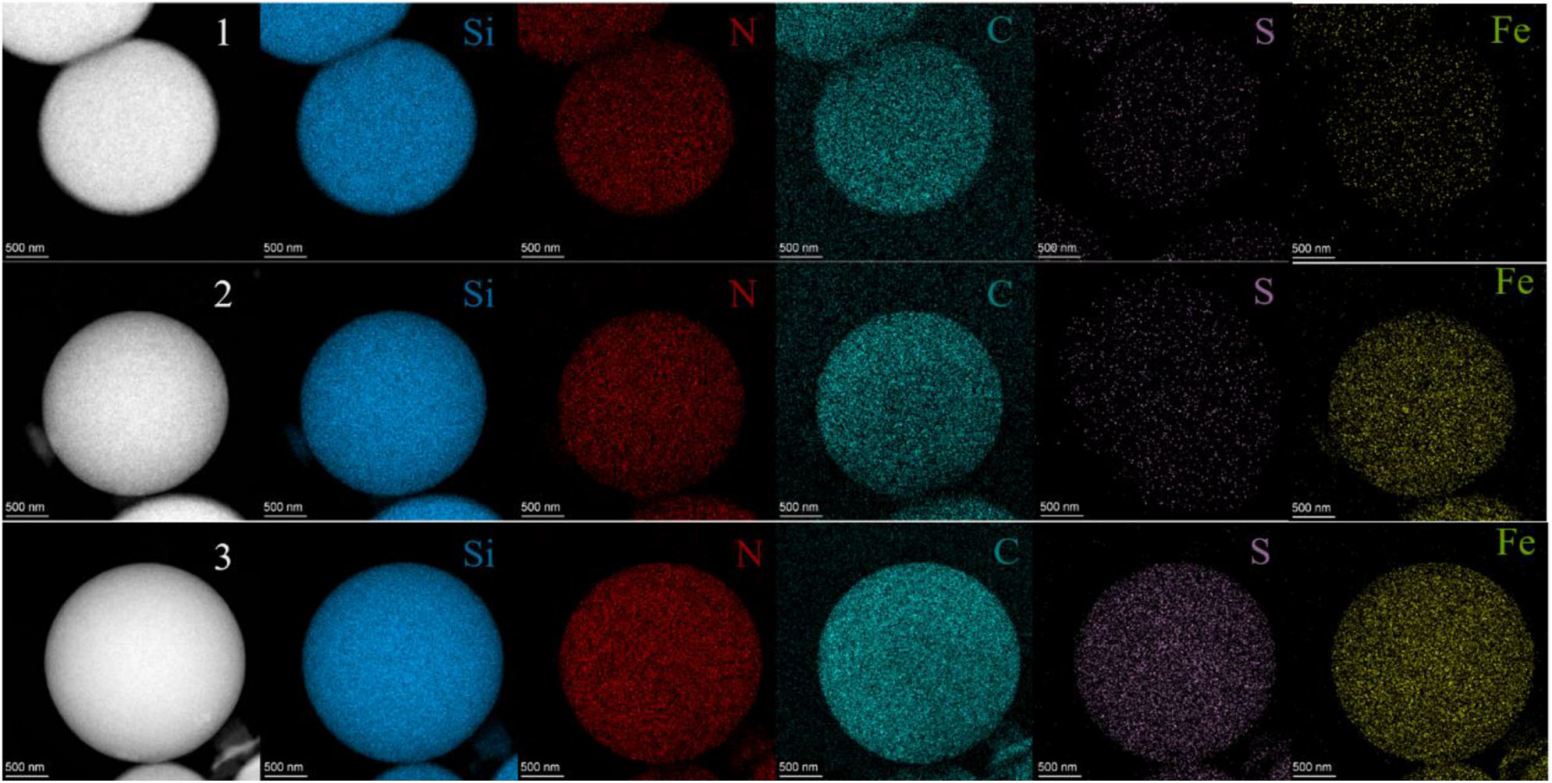
STEM images and Si, N, C, S and Fe element maps of HMSS-NH_2_ (1), HMSS-NH_2_-Fe^3+^ (2), and alcalase@HMSS-NH_2_-Fe^3+^ (3).

The SEM images of HMSS-NH_2_-Fe^3+^ were shown in Figure 1 with different magnification. It was obviously seen that HMSS-NH_2_-Fe^3+^ maintained homogeneous spherical shape and possessed similar internal cavity and surface mesoporous structure as HMSS. Meanwhile, the average diameter of HMSS-NH_2_-Fe^3+^ was determined to be 2.3 μm. In order to gain better insight into synthesized nanoparticles, the microstructure and compositional distribution of HMSS-NH_2_, HMSS-NH_2_-Fe^3+^ and alcalase@HMSS-NH_2_-Fe^3+^ were further investigated by STEM coupled with energy dispersive X-ray spectroscopy (EDX) elemental maps. The element maps of eigen element were used to perform qualitative and quantitative the compositional details of different carriers and immobilized alcalase (Chang et al., 2014). As shown in Figure 2, the TEM images and Si element mapping images verified that these as-synthesized nanoparticles possessed same spherical dense Si skeleton structure apparently. According to EDX maps and spectra of Fe, C and N element, the increase of the bright spot density in Fe element maps and significant increase of Fe peak in EDS spectra (Figure S1) of HMSS-NH_2_-Fe^3+^ providing direct evidence of the successful loading of Fe^3+^ on HMSS-NH_2_ surface. Besides, the C, N element were shown similar distribution and intensity in HMSS-NH_2_ and HMSS-NH_2_-Fe^3+^. The above result could be attributed to the same source (APTES) of C, N element in both two nanoparticles. After the immobilization of alcalase, foreseeable increase of intensity of C, N and S element in the EDS spectra of alcalase@HMSS-NH_2_-Fe^3+^ accompanied with the increasing number of bright spots representing C, N and S element were observed. The increase of C, N and S elements could be attributed to the immobilization of alcalase on HMSS-NH_2_-Fe^3+^ surface, alcalase as a protein-based biological macromolecule, which contained a large number of C, N and S elements. Furthermore, the distribution of C, N and S elements in alcalase@HMSS-NH_2_-Fe^3+^ were dense and uniform, which indicated that the immobilization of alcalase on nanoparticle surface was uniform. Besides, to further confirmed the immobilization of alcalase, alcalase@HMSS-NH_2_-Fe^3+^ was fluorescently labeled by FITC, and an extensive dialysis was performed until no release of free FITC into solution. FITC is an amine reactive fluorescent probe which labels protein by forming a covalent bond between its isothiocyanate group and the primary and secondary amine groups of lysine (Shah et al., 2019). As shown in Figure S2, the distinct green fluorescence in CLSM image of alcalase@HMSS-NH_2_-Fe^3+^ provided a persuasive confirmation for the successful immobilization of alcalase.

**Figure 3:**
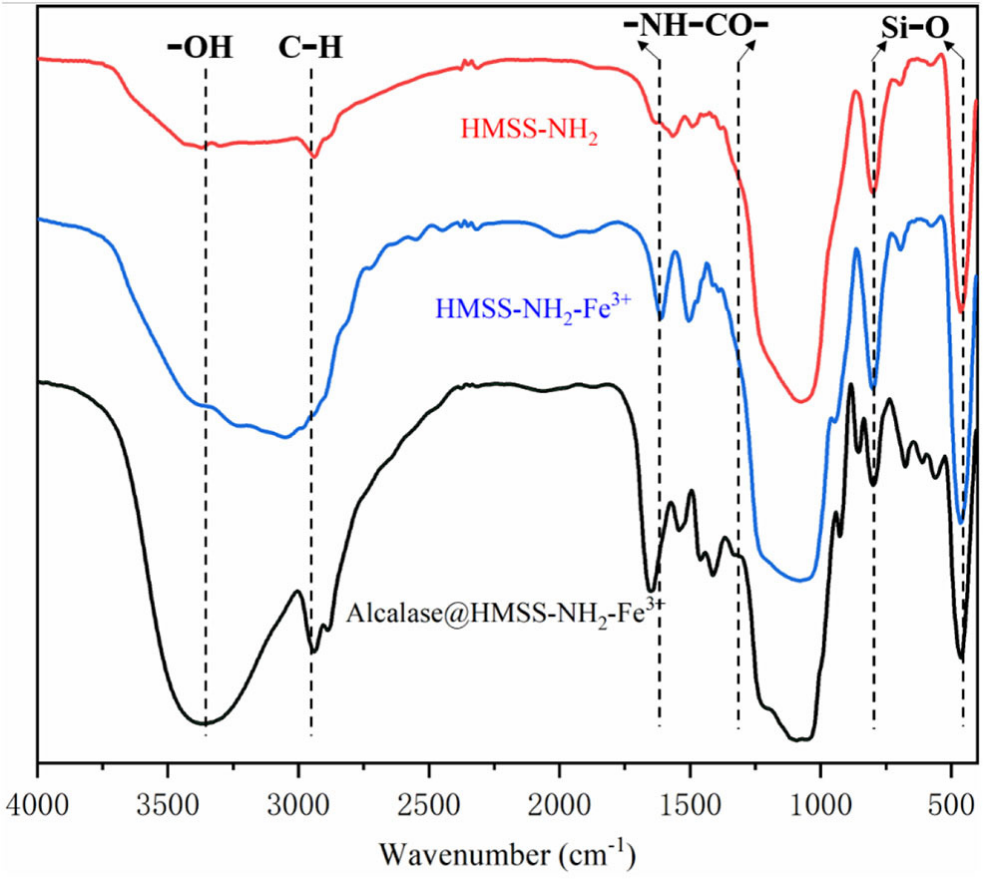
FT-IR spectra of HMSS-NH_2_, HMSS-NH_2_-Fe^3+^ and alcalase@HMSS-NH_2_-Fe^3+^.

FT-IR spectra of HMSS-NH_2_, HMSS-NH_2_-Fe^3+^, and alcalase@HMSS-NH_2_-Fe^3+^ were carried out to compare the compositions of different nanoparticles and revealed the interactions between alcalase and HMSS-NH_2_-Fe^3+^. According to Figure 3, the Si skeleton structure of matrix could be confirmed by the Si-O bending vibration at 803 cm^−1^ and Si-O symmetrical stretching vibration at 469 cm^−1^ (Al-Oweini and El-Rassy, 2009). Further, the intense Si-O covalent bonds vibrations appear mainly in the 1,250–1,000 cm^−1^ range revealing the existence of a dense silica network, where oxygen atoms play the role of bridges between each two silicon sites, which were observed in all samples (Al-Oweini and El-Rassy, 2009). The characteristic absorption peak of 3,365 cm^−1^ was ascribed to absorb hydrogen-bonded water molecules and SiO-H group on the surface of synthesized nanoparticles. Comparing with HMSS-NH_2_, the intense peaks appearing at 1,610 cm^−1^ in HMSS-NH_2_-Fe^3+^ spectra could be attributed to the stretching vibration of N-H (Bernal et al., 2018a), which indicated that the HMSS-NH_2_ was successfully modified by Fe^3+^. After alcalase immobilization, absorption peaks at 3,365 and 2,940 cm^−1^ in alcalase@HMSS-NH_2_-Fe^3+^ spectra clearly differentiated. In addition, the spectral shifting and intensity variations of protein amide I bands at 1,640 cm^−1^, amide II bands at 1,542 cm^−1^and amide III bands at 1,300 cm^−1^ were also confirmed the immobilization of alcalase.

### Enzymatic Performance Evaluation of Free and Immobilized Alcalase

**Figure 4:**
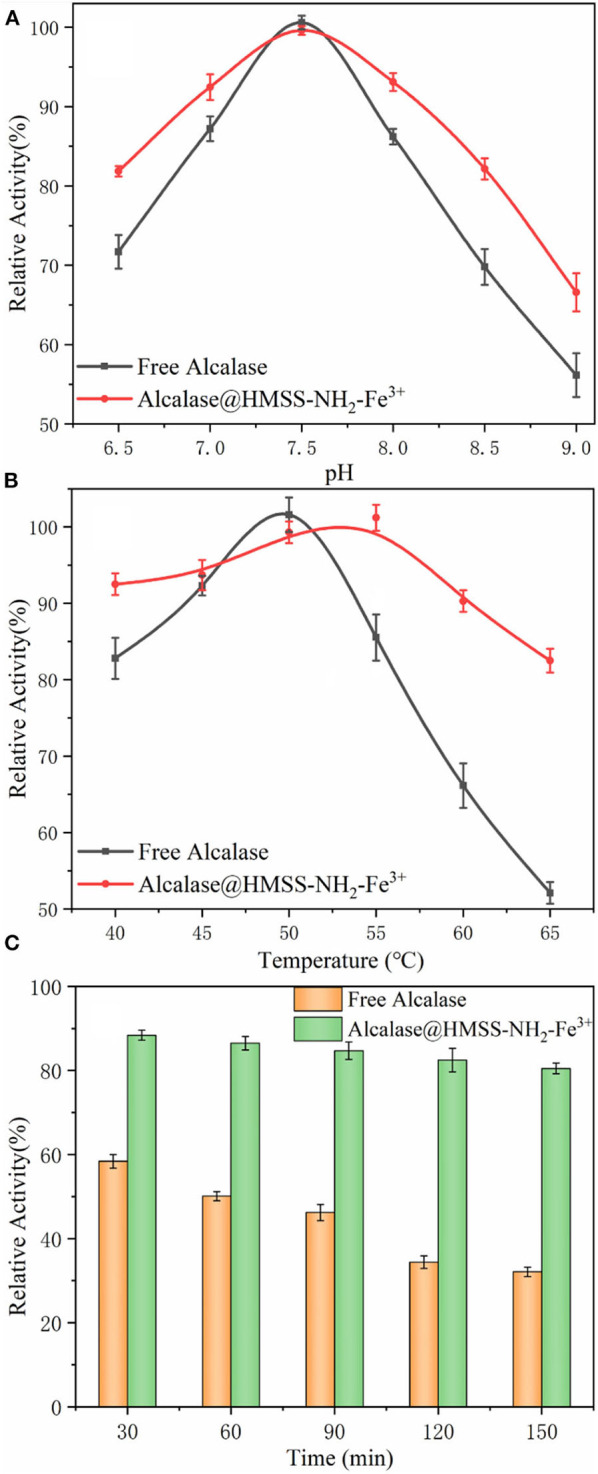
Effect of pH value **(A)** and temperature **(B)** on the activity of free alcalase and alcalase@HMSS-NH_2_-Fe^3+^. **(C)** Thermal stability of the acalase@HMSS-NH_2_-Fe^3+^ and free alcalase.

The insufficient stability of enzyme under processing conditions is the major barrier for enzyme industrial application, high temperature, or inappropriate pH value would cause the reversible or irreversible denaturation of enzyme, which would cause the losses of enzyme activity (Orfanakis et al., 2018; Liao et al., 2019). Thus, in order to check whether the prepared biocatalyst was suitable under catalytic conditions, the effects of temperature and pH value on free alcalase and alcalase@HMSS-NH_2_-Fe^3+^ were further evaluated. As shown in Figure 4, the immobilization of alcalase on HMSS-NH_2_-Fe^3+^ carriers exhibited significant influence on activity and stability of alcalase. According to Figure 4A, both alcalase@HMSS-NH_2_-Fe^3+^ and free alcalase reached maximum activity at pH 7.5, which indicated that the optimum pH of alcalase did not change after immobilization. However, the immobilization alcalase showed much higher relative activity than free alcalase both in acidic and basic pH regions. For example, the relative activities of free alcalase were 71.7 ± 2.1% and 56.2 ± 2.8% at pH 6.5 and 9.0, respectively. Under the same pH condition, the relative activities of alcalase@HMSS-NH_2_-Fe^3+^ were 81.9 ± 0.6% and 66.6 ± 2.4%, respectively. The increase of pH stability frequently associated with proton donor or acceptor groups (hydroxy, amino ect.) on the support, which could adjust the pH of surrounding environment of immobilization enzyme (Bayramoglu et al., 2013). Thus, it implied that alcalase@HMSS-NH_2_-Fe^3+^ had a much stronger pH tolerance than the free alcalase, which would reduce the losses of enzyme activity caused by inappropriate pH condition during the practical application.

In addition, thermal stability was also a crucial factor in the application of alcalase (Cardelle-Cobas et al., 2016). As shown in Figure 4B, the alcalase@HMSS-NH_2_-Fe^3+^ exhibited higher optimum temperature (55°C) than free alcalase (50°C). This change of optimum temperature could be due to the coordinate covalent bonds formation between alcalase and support caused by pre-coordinated Fe^3+^ on support, which might increase the conformational inflexibility of enzyme and prevent it from distortion or dissociation by heat exchange (Hu et al., 2015). Furthermore, alcalase@HMSS-NH_2_-Fe^3+^ showed more stable performance than free alcalase. Alcalase@HMSS-NH_2_-Fe^3+^ maintained high relative activity above 82.5 ± 1.1% in the temperature range of 40°C to 65 °C. However, the relative activity of free alcalase was decreased from 100 ± 0.8% to 52.1 ± 1.4% with the increase of temperature from 50 to 65 °C. Meanwhile, the study of thermal stability of free and immobilized alcalase showed a similar result. The alcalase@HMSS-NH_2_-Fe^3+^ retained 80.5 ± 2.4% initial activity after incubation at 85°C for 150 min. However, the residual activity of free alcalase decreased to 46.2 ± 4.2% after treated at 85°C for 60 min (Figure 4C). The higher thermal tolerance might be attributed to the protection of favorable carrier (HMSS) and metal-protein affinity immobilization strategy. These above results revealed that acalase@HMSS-NH_2_-Fe^3+^ exhibited more stable enzymatic performance at harsh conditions than free alcalase.

### Kinetic Analysis of the Amidase Activity of Free Alcalase and Alcalase@HMSS-NH_2_-Fe^3+^

**Figure 5:**
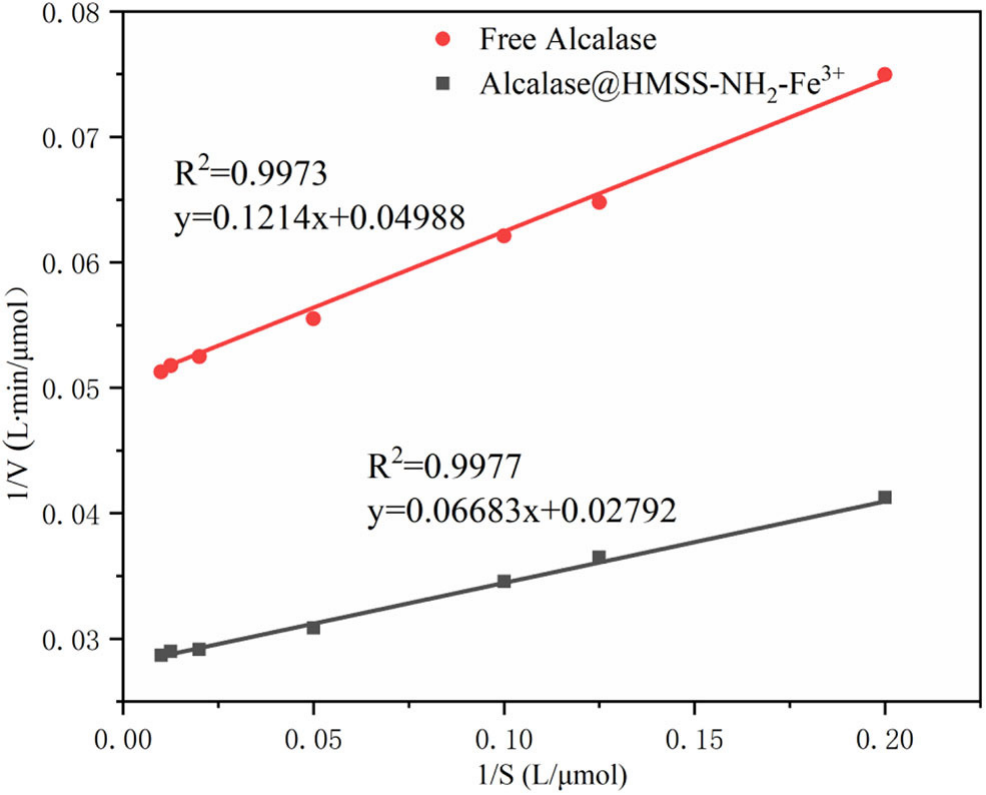
Lineweaver-Burk double reciprocal plots of free alcalase and acalase@HMSS-NH_2_-Fe^3+^.

The optimal conditions for the free alcalase and alcalase@HMSS-NH_2_-Fe^3+^ were used to study the kinetic parameters. The Lineweaver-Burk double reciprocal plot of free and immobilization alcalase was shown in Figure 5 and Table 2. From the calculated data, the K_m_ for alcalase@HMSS-NH_2_-Fe^3+^ was 2.3 μmol/L, which was lower than the free alcalase (2.4 μmol/L). This finding suggested that the affinity between the substrate and the acalase@HMSS-NH_2_-Fe^3+^ was higher than that of free alcalase (Zhang et al., 2017). Similarly, compared to free state, the V_max_ of acalase@HMSS-NH_2_-Fe^3+^ was shown a remarkable increase, from 20.0 to 35.8 μmol/(L·min). The apparent kinetic constant V_max_/K_m_ of alcalase@HMSS-NH_2_-Fe^3+^ (15.6 min^−1^) was 1.9-fold higher than that of free alcalase (8.3 min^−1^). Those significant (P < 0.05) increases of catalytic performance could be attributed to multiple factors. Firstly, highly ordered frameworks of HMSS-NH_2_-Fe^3+^ provided a uniform and relatively independent microenvironment for alcalase to defense extremely harsh conditions and decreased the possibility of autolysis. Secondarily, the large specific surface of HMSS-NH_2_-Fe^3+^ provided an essential prerequisite for efficient mass transfer during the catalysis process. Last but not least, the binding of alcalase with pre-coordination Fe^3+^ resulted in the favorable change in conformation of the alcalase, which enhanced the catalytic performances of immobilized alcalase.

**Table 2:** Kinetics parameters of the free and immobilized lipase.

	**Vmax (mol/(L.min))**	**Km (mol/L)**	**Vmax/Km (min^**−1**^)**
Free alcalase	20.0	2.4	8.3
Acalase@HMSS-NH_2_-Fe^3+^	35.8	2.3	15.6

### Effect of Metal-Protein Affinity Immobilization on the Structure and Activity of Alcalase

**Figure 6:**
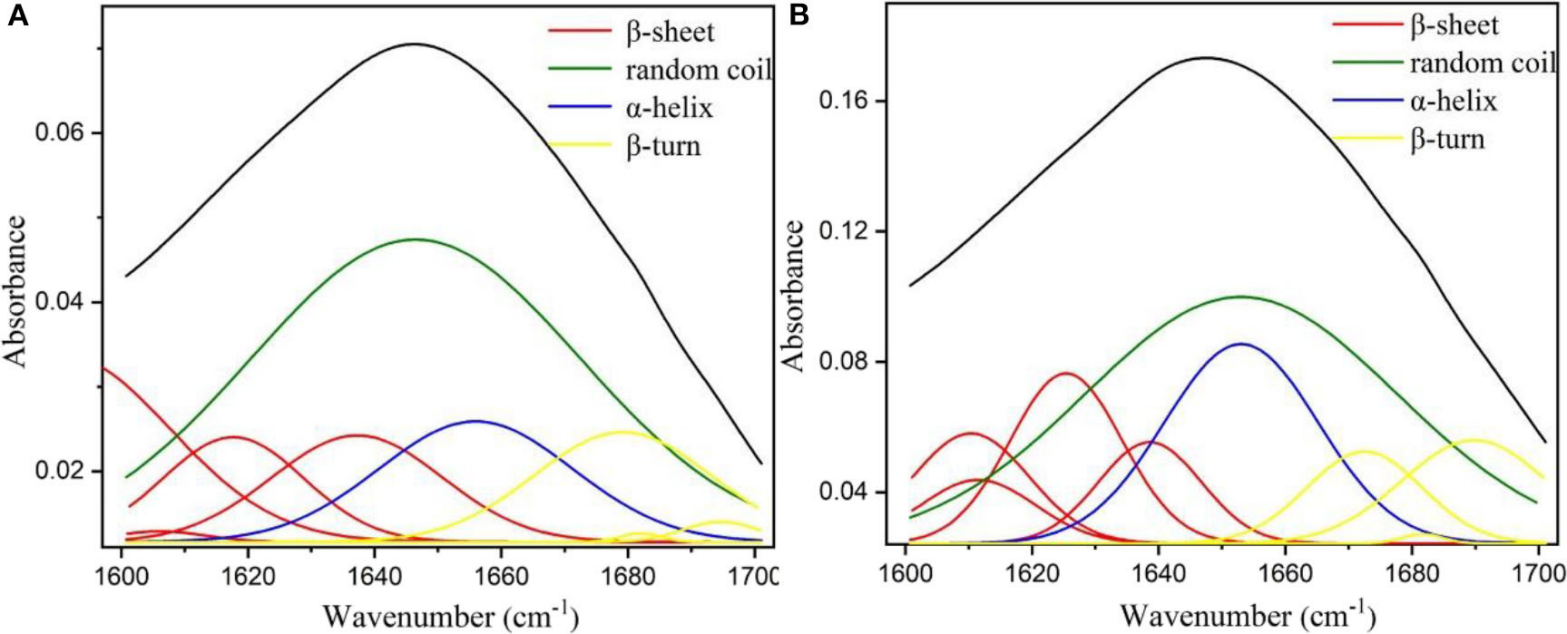
Fourier transform infrared (FT-IR) spectra of **(A)** free alcalase and **(B)** immobilized alcalase by Gaussian multi-peak fitting.

The aforementioned results have indicated that metal-protein affinity immobilization strategy could significantly enhance the biocatalytic activity and stability of alcalase. These enhancements of enzymatic performance could be attributed to the changes of secondary structure of alcalase (Greener and Sternberg, 2018), because the active site of alcalase was formed at the interface between α and β-domains in its secondary structure (Ma et al., 2011). Thus, in order to provide intuitive evidence for the enzymatic activation and stabilization of metal-protein affinity immobilization strategy, the free alcalase and alcalase@HMSS-NH_2_-Fe^3+^ FTIR spectra of the amide I region (1,700–1,600 cm^−1^) were further determined based on the Gaussian multi-peak fitting. Figure 6 showed the Gaussian multi-peak fitting results of amide I region (1,700–1,600 cm^−1^) of free alcalase and alcalase@HMSS-NH_2_-Fe^3+^, the band located at 1,610–1,640 cm^−1^, 1,640–1,650 cm^−1^, 1,650–1,658 cm^−1^ and 1,660–1,700 cm^−1^ was assigned to β-sheet, random coil, α-helix, and β-turn content, respectively (Qi et al., 2019). Furthermore, the contributions of each fraction of secondary structure were summarized in Table 3.

**Table 3:** Fraction of secondary structures and amidase activity of free alcalase and alcalase@HMSS-NH_2_-Fe^3+^.

**Secondary structure**	**α-helix (%)**	**β-sheet (%)**	**β-turn (%)**	**Random coil (%)**	**Amidase activity (U/mg)**
Free alcalase	12.9 ± 0.1	24.1 ± 0.2	11.1 ± 0.1	51.9 ± 0.3	10.5 ± 2.7
Alcalase@HMSS-NH_2_-Fe^3+^	17.1 ± 0.1	28.7 ± 0.1	13.1 ± 0.1	41.1 ± 0.1	55.5 ± 6.2

According to the results of Figure 6 and Table 3, the immobilization of alcalase based on metal-protein affinity immobilization strategy significantly changed the secondary structure of alcalase, which resulted in the increased amidase activity of alcalase@HMSS-NH_2_-Fe^3+^, from 10.5 ± 2.7 to 55.5 ± 6.2 U/mg. In free state, alcalase showed a loose structure, manifested as high random coil content, up to 51.9 ± 0.3%. The environmental sensitivity of alcalase (temperature, pH value etc.) was caused by the loose secondary structure, which was mentioned in Figure 4. After immobilization, a notable reduction of random coil content was observed, from 51.9 ± 0.3% to 41.1 ± 0.1%. Meanwhile, the contents of α-helix (12.9 ± 0.1 to 17.1 ± 0.1%), β-sheet (24.1 ± 0.2 to 28.7 ± 0.1%) and β-turn (11.1 ± 0.1 to 13.1 ± 0.1%) were increased in varying degrees. The above results indicated that the alcalase after immobilization possess more regularity and flexibility secondary structure than free alcalase, which caused the significant enhancement of biological activity and stability of alcalase.

### Proteolysis Activity of Free and Immobilized Alcalase

**Figure 7:**
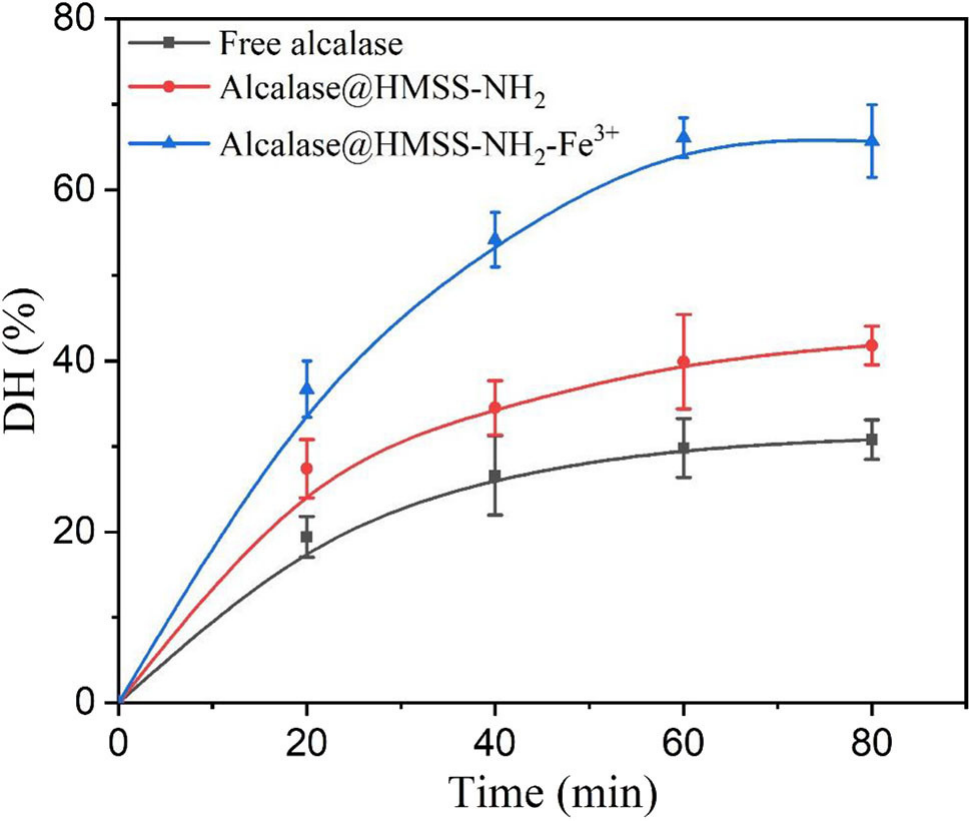
Hydrolysis degree of BSA with free alcalase, alcalase@HMSS-NH_2_ and alcalase@HMSS-NH_2_-Fe^3+^ as the biocatalyst. Hydrolysis conditions: biocatalysts: BSA =1:10 (w/w), 40°C, pH 7.5.

Proteolysis, as an important biocatalysis process, was widely used in the food industry and proteome analysis (Zhang et al., 2019). The albumin from bovine serum (BSA) was adopted as a model protein to evaluate the proteolysis activity of alcalase@HMSS-NH_2_-Fe^3+^, alcalase@HMSS-NH_2_ and free alcalase under the same conditions. As shown in Figure 7, alcalase@HMSS-NH_2_-Fe^3+^ exhibited highest proteolysis efficiency for BSA in 60 min. The hydrolysis degree (DH) of BSA reached 65.7 ± 4.2% at the proteolysis terminal point which was 1.5-fold and 2.1-fold higher than that of alcalase@HMSS-NH_2_ and free alcalase, respectively. The outstanding proteolysis performance of alcalase@HMSS-NH_2_-Fe^3+^ could be ascribed to the combination of favorable carrier (HMSS) and metal-protein affinity immobilization strategy. The immobilization of alcalase on HMSS-NH_2_-Fe^3+^ not only provided high-efficiency mass transfer and proper microenvironment for proteolysis, but also favored the activity and stability of by changing the secondary structure of alcalase.

In addition, the molecular weight (MW) distribution of hydrolysates produced by three forms of biocatalyst visually demonstrated the outstanding catalytic performances of alcalase@HMSS-NH_2_-Fe^3+^. According to Figure S3, the complete disappearance of BSA band in lane.1 indicated that alcalase@HMSS-NH_2_-Fe^3+^ hydrolyzed all BSA (MW: 66.4 kDa) into smaller molecular weight components. And at the same processing time, alcalase@HMSS-NH_2_ and free alcalase only hydrolyzed 62.2 and 23.2% of BSA, respectively.

### Comparisons of Recycle of Different Immobilized Alcalase

**Figure 8:**
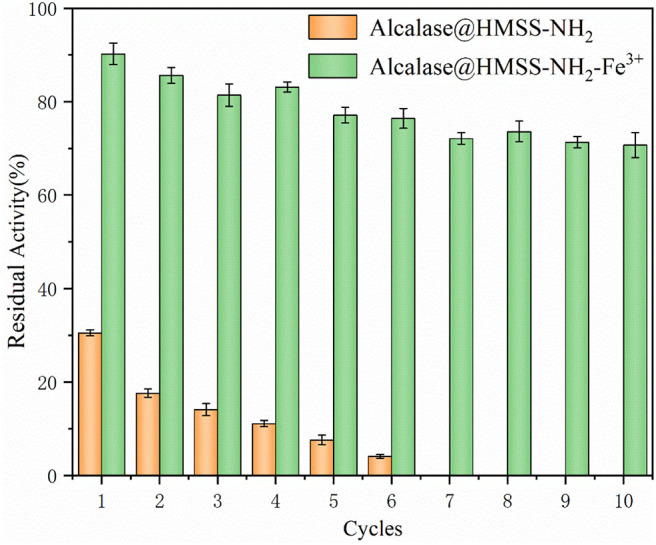
The reusability of acalase@HMSS-NH_2_-Fe^3+^ and acalase@HMSS-NH_2_. The reaction was incubated at 40°C, pH 7.5 for 5 min with 3 mL BAEE (10 mM) as substrate.

The reusability of alcalase@HMSS-NH_2_-Fe^3+^ and alcalase @HMSS-NH_2_ was compared to investigate the potentiality of these biocatalysts in industrial applications. Commonly, there are three main factors, which would cause the deactivation of immobilized enzyme over industrial applications: the presence of frictional shear in batch stirring reactions, incomplete recovery and extreme environmental factors. These factors could cause the inactivation and leakage of enzyme, thus result in the losses of enzymatic activity. As shown in Figure 8, it could be concluded that the residual activity remained up to 70.7 ± 3.7% after 10 cycles, but the alcalase@HMSS-NH_2_ was only retained 4.1 ± 2.1% of its initial activity after 6 cycles. In comparison, the immobilized alcalase on amino-functionalized Fe_3_O_4_ nanoparticles (Hu et al., 2015) lost more than 50% of its initial activity after 10 cycles; similarly, the alkaline protease nanoflowers-PVA composite hydrogel (Zhang et al., 2018) only retained 18.2% of its initial activity after 10 cycles. Based on these results, it could be deduced that the alcalase microarray based on metal-protein affinity immobilization method could efficiently reduce the bioactivities losses of alcalase caused by enzyme leaking and autolysis during the catalytic process.

## Conclusions

In this study, a novel alcalase microarray (alcalase@HMSS-NH_2_-Fe^3+^) with excellent biocatalytic performance was successfully constructed based on metal ion modified amino-functionalized HMSS. The tailor-made nanocatalyst combined the advantages of HMSS and metal-protein affinity immobilization strategy, which endowed ultra-high activity and exceptional stability for alcalase@HMSS-NH_2_-Fe^3+^. The novel immobilization strategy based on metal-protein affinity enhanced the activity and stability of alcalase@HMSS-NH_2_-Fe^3+^ *via* changing the conformation of the secondary structure of alcalase. Compared with the free alcalase, the amidase activity and hydrolysis efficiency of alcalase@HMSS-NH_2_-Fe^3+^ increase by 5.3-fold and 2.1-fold, respectively. The strategy could enhance the reusability of alcalase@HMSS-NH_2_-Fe^3+^ greatly and the alcalase microarray still retained most of its initial activity after 10 cycles of successive reuse. In conclusion, this study established a promising strategy to overcome the disadvantages posed by free alcalase, which provided new expectations for the application of alcalase in sustainable and efficient proteolysis.

## Data Availability Statement

All datasets generated for this study are included in the article/Supplementary Material.

## Author Contributions

QZ carried out the experiments. QZ and MZ conceived and designed the experiments. QZ, DS, QL, and MZ analyzed the data and wrote the manuscript.

## Conflict of Interest

The authors declare that the research was conducted in the absence of any commercial or financial relationships that could be construed as a potential conflict of interest.
