# Supplementary material for: Alcalase Microarray Base on Metal Ion Modified Hollow Mesoporous Silica Spheres as a Sustainable and Efficient Catalysis Platform for Proteolysis
Source: Front Bioeng Biotechnol. 2020 Jun 10;8:565. doi: 10.3389/fbioe.2020.00565 (PMC7297948; doi:10.3389/fbioe.2020.00565)
Supplement: Supplementary file 1 [file Image_1.pdf]

Supplementary Material

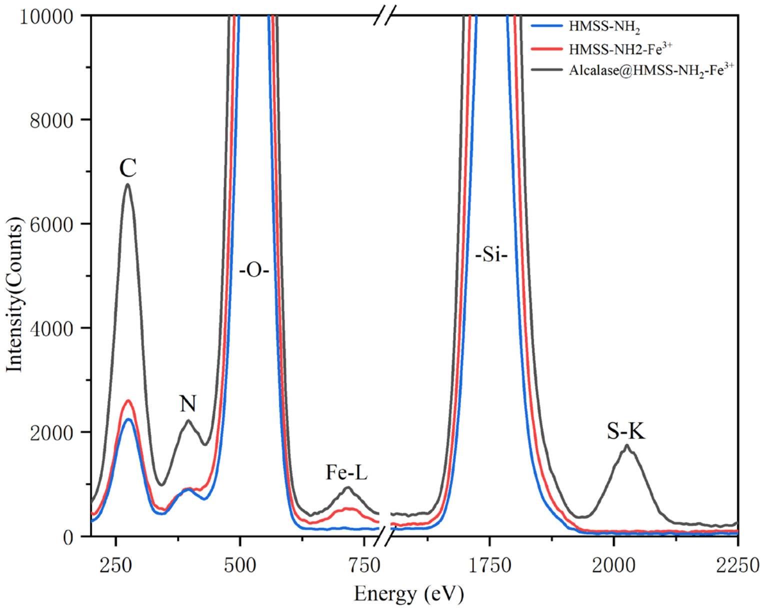

FIGURE S1. EDX spectra of $HMSS\text{-}NH_2$, $HMSS\text{-}NH_2\text{-}Fe^{3+}$, $Alcalase@HMSS\text{-}NH_2\text{-}Fe^{3+}$.

FIGURE S2. Confocal laser scanning microscopy (CLSM) images of FITC-labeled alcalase@HMSS-NH_2-Fe^{3+}.

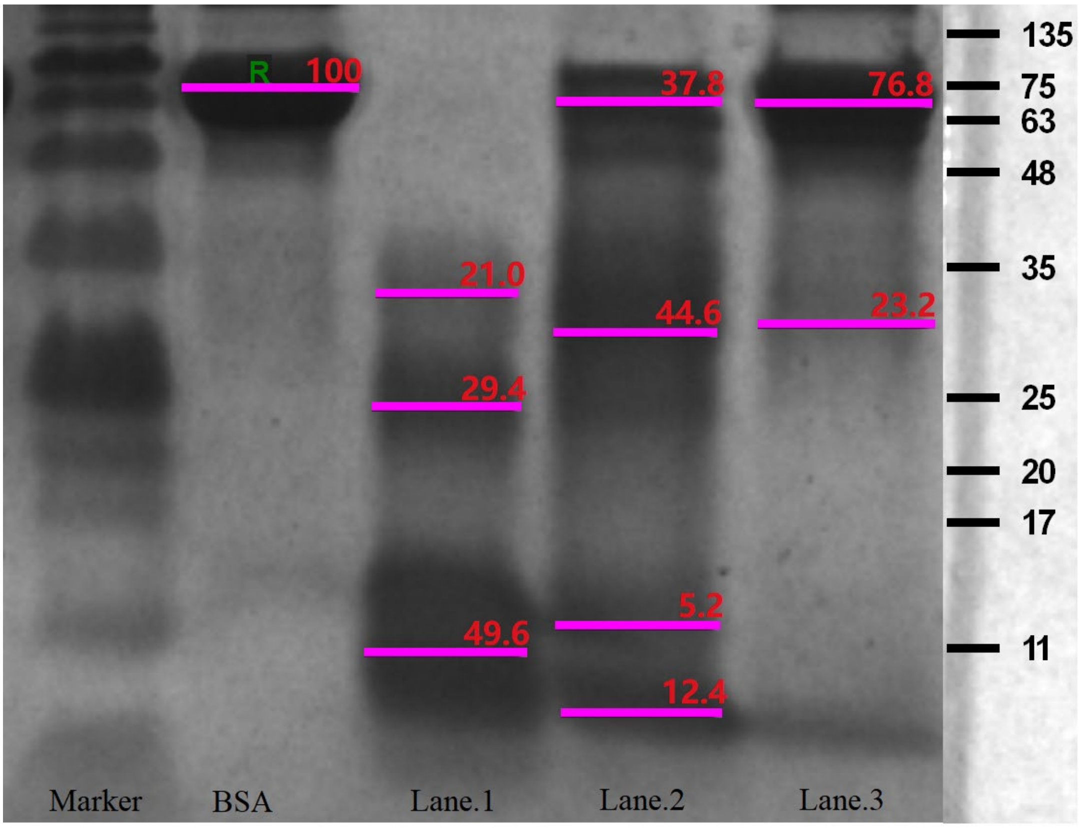

FIGURE S3. Molecular weight (MW) distribution of BSA hydrolysates produced by alcalase@HMSS-NH_2-Fe^{3+}, alcalase@HMSS-NH_2 and free alcalase. Lane. 1: BSA hydrolysates with alcalase@HMSS-NH_2-Fe^{3+} at 40 ℃ for 15 min, Lane. 2: BSA hydrolysates with alcalase@HMSS-NH_2 at 40 ℃ for 15 min, Lane. 3: BSA hydrolysates with free alcalase at 40 ℃ for 15 min.
